# NCAPG facilitates colorectal cancer cell proliferation, migration, invasion and epithelial–mesenchymal transition by activating the Wnt/β-catenin signaling pathway

**DOI:** 10.1186/s12935-022-02538-6

**Published:** 2022-03-15

**Authors:** Yanlong Shi, Chang Ge, Debao Fang, Wei Wei, Li Li, Qian Wei, Hongzhu Yu

**Affiliations:** 1 Department of General Surgery, Fuyang Hospital Affiliated to Anhui Medical University, Fuyang, 236000 Anhui China; 2 School of Life Sciences and Medical Center, University of Science & Technology of China, Hefei, 230000 Anhui China; 3 School of Nursing, Anhui Medical University, HeFei, 230000 Anhui China

**Keywords:** NCAPG, Colorectal cancer, Epithelial–mesenchymal transition, Wnt/β-catenin signaling pathway

## Abstract

**Background:**

The condensation complex gene non-SMC condensin I complex subunit G(NCAPG), a cell cycle-associated condensin, is over-expressed in various cancers. However, its biological function in colorectal cancer (CRC) has yet to be deciphered. In this study, we investigated the role of NCAPG in CRC progression.

**Methods:**

Tissues and cells were used to measure NCAPG expression levels and their association with clinicopathological characteristics. NCAPG silencing and overexpression in CRC cells were used to measure its effect on proliferation, migration, invasion, and epithelial–mesenchymal transition (EMT) progression. In addition, mRNA, and protein expression levels of key EMT biomarkers were measured. The underlying mechanism of NCAPG modulating CRC progression was further explored using western blotting, co-immunoprecipitation (CO-IP), and immunofluorescence (IF) assays.

**Results:**

NCAPG was over-expressed in CRC tissues and cell lines. High expression levels were associated with differentiation levels, lymph metastasis, and vascular invasion in patients. NCAPG silencing suppressed, while NCAPG overexpression promoted the proliferative, migration, and invasive capacity of HCT116 and SW480 cells. Mechanistically, we discovered that NCAPG participated in regulating the EMT process and the Wnt/β-catenin signaling pathway to facilitate CRC invasion and metastasis. Additional experiments demonstrated that NCAPG activated the Wnt/β-catenin signaling pathway by binding to β-catenin in CRC cells.

**Conclusion:**

NCAPG acts as an oncogene involved in the development and progression of CRC by binding to β-catenin to activate the Wnt/β-catenin signaling pathway.

## Background

Colorectal cancer (CRC) is a common gastrointestinal malignancy worldwide. According to the latest global cancer statistics, the worldwide morbidity and mortality of CRC are the 3rd and 2nd worldwide respectively, and is a significant challenge to global public health [1]. Notably, the incidence of CRC in younger individuals has gradually increased [2]. At present, the prognosis of CRC patients has been greatly improved. The survival rate for patients with stage I and stage II CRC are 91% and 82%, respectively. However, the survival rate of patients diagnosed with stage IV disease is only 12% [3]. During the occurrence and progression of CRC, there are biological changes such as protooncogene activation, tumor suppressor gene inactivation, and DNA repair-related gene mutations [4, 5]. However, reliable biomarkers to monitor these biological changes are limited. Hence, it is vital to identify CRC-related genes and their functions, not only to understand their molecular mechanism but to identify patients early. These biomarkers will help with clinical diagnosis, treatment, and prognosis.

Non-SMC condensin I complex subunit G (NCAPG) is a condensin complex subunit encoded by the NY-MEL-3 gene located on human chromosome 4p15.32, which is mainly responsible for chromosome cohesion and stability during mitosis and meiosis [6]. Growing evidence has indicated that aberrant NCAPG expression is strongly associated with various cancers, such as liver [7], renal cell [8], prostate [9], and breast cancer [10]. NCAPG is involved in cell cycle progression by modulating the PI3K/AKT signaling pathway [11]. Results from next-generation sequencing comparing expression levels from tumor and adjacent normal tissues have suggested that NCAPG could be used as a prognostic biomarker for liver and lung adenocarcinoma [12, 13]. However, the biological function of NCAPG as it relates to CRC had not been fully elucidated.

The Wnt/β-catenin signaling pathway is an evolutionarily conserved and unique signaling pathway that has a significant role in embryonic development, tissue self-renewal, and homeostasis [14]. The hyper-activation of the Wnt signaling pathway directly induces the occurrence of CRC [15]. Mutations in APC or β-catenin, which are important members of the Wnt signaling pathway, induces downstream transcriptional activation, resulting in cell division and tumor growth [16, 17]. β-Catenin is a core factor of the Wnt signaling pathway and bridges the Wnt signaling pathway with epithelial–mesenchymal transformation (EMT). The EMT process increases the migration and invasion of cancer cells, which is considered as one of the primary reasons for recurrence and metastasis [18]. Furthermore, recent studies have revealed the crucial significance of EMT in the modulation of CRC diagnosis and prognosis [19, 20]. The activation of the Wnt/β-catenin signaling pathway has been demonstrated to play a vital role in EMT required for CRC metastasis [21]. In addition, the Wnt/β-catenin signaling pathway facilitates EMT by increasing Snail expression [22]. However, whether NCAPG associates with β-catenin to activate the Wnt/β-catenin signaling pathway needs to be determined.

We found that NCAPG was up-regulated in CRC tissues and cells and was correlated with clinicopathological features. Functionally, we demonstrated that lentivirus mediated NCAPG silencing, or overexpression affected proliferation, migration, invasion and EMT of CRC cells. Furthermore, we demonstrated that NCAPG could activate the Wnt/β-catenin signaling pathway by binding to β-catenin. Our findings would be valuable for the clinical diagnosis and treatment of CRC.

## Materials and methods

### Public database assay

UALCAN is an interactive database based on The Cancer Genome Atlas (TCGA) RNA-seq and clinical data of 31 cancer types [23]. Oncomine database is currently the largest data mining platform worldwide, which aims to explore novel biomarkers or potential therapeutic targets. Differential expression analysis was used to measure NCAPG mRNA levels in CRC tissues and normal tissues from the Hong colorectal Statistic [24]. Using UALCAN as well as the Linked Omics database, we not only validated the expression of NCAPG in different cancers but also analyzed the clinicopathological characteristics in CRC [25]. TIMER2.0 database provides Immune Association, Cancer Exploration and Immune Estimation modules, and can explore the association with cancer in the TCGA cohort [26]. TIMER 2.0 was used to estimate the linkage between NCAPG and CTNNB1 expression in CRC.

### Tissue samples

91 pairs of CRC tissue and adjacent non-cancerous tissues from patients were collected from the Department of General Surgery, Fuyang Hospital Affiliated to Anhui Medical University after informed consent. The study was approved by the Ethics Committee of Fuyang hospital affiliated Anhui Medical University.

### Cell culture

Human CRC cell lines (HCT116, SW480, HT-29) and human normal colonic epithelial cells (NCM460) were purchased from Cell Bank (Shanghai, China) and Procell Life Science (Wuhan, China). Cells were cultured in DMEM (HyClone) with high-glucose and glutamine containing 10% fetal bovine serum (VivaCell, Shanghai, China) and 1% Penicillin/Streptomycin (Gibco, Unite States). Cells were incubated in a humidified atmosphere of 5% CO_2_ at 37 °C.

### Immunohistochemistry

91 pairs of CRC tissues and adjacent tissues were dehydrated and paraffin-embedded. The paraffin blocks tissues were sliced into 4 µm thick sections and then baked overnight at 60 °C, followed by conventional dewaxing and antigen retrieval. Tissue sections were infiltrated with NCAPG primary antibody (1:200; Abcam, Cambridge, UK) at 4 °C overnight. Sections were then washed and incubated with horseradish peroxidase-conjugated for 30 min. After staining, the sections were counter-stained with hematoxylin. Two investigators read and scored the tissues independently.

### Lentivirus transduction

NCAPG (NM_022346) shRNA lentivirus (LV-shNCAPG1), shRNA lentivirus vector (LV-shNCAPG NC), NCAPG lentivirus (LV-NCAPG), and negative control (LV-NCAPG NC) were purchased from GeneChem (Shanghai, China). The target sequence of NCAPG was 5′-GCCTTAACAGTACATGACAAT-3′ for shNCAPG1, 5′-CGGGCAGTGTTATCATGTATT-3′ for LV-shNCAPG2; 5′-CAAACAGCAATGCAGTGAGAT-3′ for LV-shNCAPG3, and 5′-TTCTCCGAACGTGTCACGT-3′ for shRNA-NC. Stable cell lines were generated under puromycin selection. Knockdown and overexpression efficiency was determined by qRT-PCR and Western blotting.

### Quantitative real-time PCR (qRT-PCR)

Total RNA was extracted from tissues and cells using TRIzol reagent (Takara). To quantify NCAPG levels, reverse transcription of cDNA was performed using primescript™ rt kit (Takara). NCAPG expression levels were measured using SYBR Green qPCR Mix (Takara). The primers used were as follows: GAPDH forward: 5′-CTCACCGGATGCACCAATGTT-3′ and GAPDH reverse, 5′-CGCGTTGCTCACAATGTTCAT-3′; NCAPG forward, 5′-ATTGCTTTGTATTGGTGTGCCCTTTG-3′ and NCAPG reverse, 5′-ACAACTGGAATGCTCTGGATGTAACTC-3′; E-cadherin forward, 5′-GATTCTGCTGCTCTTGCTGTTTCTTC-3′ and E-cadherin reverse, 5′-GGTCCTCTTCTCCGCCTCCTTC-3′; N-cadherin forward, 5′-CTTGTGCTGATGTTTGTGGTATGGATG-3′ and N-cadherin reverse, 5′-AGTCATAGTCCTGGTCTTCTTCTCCTC-3′; Vimentin forward, 5′-TCGTGAATACCAAGACCTGCTCAATG-3′ and Vimentin reverse, 5′-ACAACTGGAATGCTCTGGATGTAACTC-3′; c-Myc forward, 5′-CTGAGGAGGAACAAGAAGATGAGGAAG-3′ and c-Myc reverse, 5′-TCCAGCAGAAGGTGATCCAGACTC-3′; CyclinD1 forward: 5′-GCCCTCGGTGTCCTACTTCAAATG-3′ and CyclinD1 reverse, 5′-TCCTCCTCGCACTTCTGTTCCTC-3′; The relative expression levels of NCAPG was determined using the 2^−ΔΔCt^ method.

### Cell proliferation assay

The cell counting kit-8 (CCK-8; DOJINDO, Japan) was evaluated cell proliferation following the manufacture’s protocol. Following cell transfection, 3 × 10^3^ cells were incubated in 96-well plates overnight. After 0, 24, 48, and 72 h, 10 µL of CCK-8 solution was added to each well and incubated at 37 °C for 2 h. Absorbance was determined at OD450 and used to determine cell proliferation.

### Transwell assays

Migration and invasion of lentivirus transfected cells were determined using Transwell Assays. Migration and invasion in the presence or absence of Matrigel™ (Corning, NY, USA) in transwell chambers (8 µm; Costar, New York, NY, USA). Briefly, 3 × 10^4^ transfected cells were resuspended in 200 µL of serum-free DMEM medium adding to the upper chamber, then 600 µL of DMEM medium containing 20% FBS was added to the lower chamber. To assess invasion ability, the upper chamber was coated with 50 µL of Matrigel. After 24 h, the migrated and invasive cells were fixed in 4% paraformaldehyde for 30 min, followed by staining with 1% crystal violet for 10 min. Cell numbers were determined and photographed using an inverted microscope. Five randomly selected areas per well were used for calculating cell numbers.

### Western blotting

Lentivirus transfected cells were lysed on ice with RIPA lysate (Beyotime, China) supplemented with protease and phosphatase inhibitors. BCA protein detection kit (Beyotime, China) was used to measure protein concentration in the supernatants. Sample extracts were loaded onto a 10% denaturing SDS-PAGE gel, electrophoresed, and then transferred to a 0.45 µm polyvinylidene fluoride (PVDF; Beyotime, China) membrane. The PVDF membranes were blocked with 5% non-fat milk at room temperature for 2 h, and then incubated with specific primary antibodies at 4 °C overnight followed by incubation with either a mouse or rabbit secondary antibody conjugated to HRP. Specific protein bands were visualized using the enhanced chemiluminescent (ECL) assay kit (Thermo Scientific, PA, USA). The following primary antibodies were used for western blotting, NCAPG (ab226805), E-cadherin (ab231303), Vimentin (ab137321), β-catenin (ab32572), GSK-3 (ab32391), p-β-catenin (ab75777), c-Myc (ab32072) and CyclinD1 (ab16663). All antibodies were purchased from Abcam.

### Immunofluorescence assays

Lentivirus transfected cells were fixed with paraformaldehyde for 20 min, permeabilized with Triton X-100, and blocked with goat serum for 30 min. Following incubation with rabbit anti-β-catenin (1:200) at 4 °C overnight, the cells were incubated with Alexa Flour-conjugated goat anti-rabbit secondary antibody (1:300; Bioss, China) for 1 h in the dark. Cells were then incubated with DAPI, and fluorescence images were captured using a laser confocal microscope.

### Co-immunoprecipitation (CO-IP) assays

Cells were lysed in RIPA lysis buffer (Beyotime) containing protease inhibitors. Cell extracts were then incubated with NCAPG or β-catenin antibodies at 4 °C overnight. Immune complexes were captured using protein A/g agarose beads and subsequently used for immunoblot analysis. IgG antibody served as the negative control for IP.

### Statistical analysis

All statistical analyses were performed using SPSS Version 22.0 software. GraphPad Prism Version 8.0 software was used to generate graphs. Descriptive statistics were used to summarize the baseline characteristics of clinicopathology. Chi-square test was used for categorical variables, Mann–Whitney U test for continuous variables, and the analysis of three or more groups was performed using one-way ANOVA. Results were generated using three independent experiments and were presented as mean ± standard deviation (SD). P < 0.05 denoted statistical significance.

## Results

### NCAPG is overexpressed in CRC tissues and cells

**Fig. 1:**
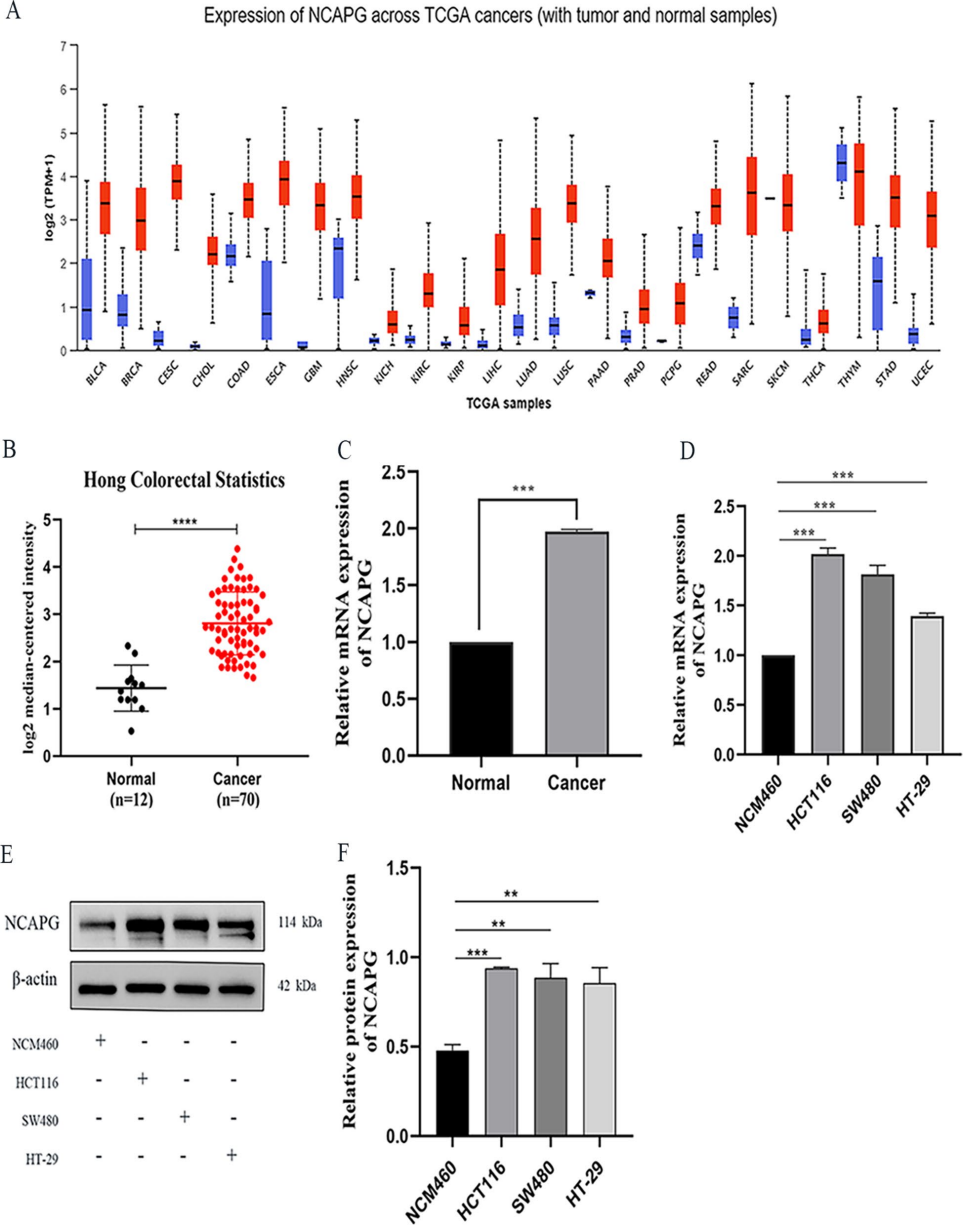
Upregulation of NCAPG expression in CRC tissues and cells. **A** Expression of NCAPG across TCGA cancers (with tumor and normal samples) in UALCAN database. **B** Expression of NCAPG in Hong Colorectal Statistics based on Oncomine database. **C** The mRNA expression of NCAPG in 60 pairs of CRC tissues and adjacent normal tissues by qRT-PCR. **D**–**F** NCAPG expression in in colon epithelial cell and CRC cell lines was detected by qRT-PCR and Western blotting analysis. *P < 0.05, **P < 0.01, ***P < 0.001 and ****P < 0.0001

The expression levels of NCAPG in each tumor were obtained from the UANCLAN database, which indicated that NCAPG was up-regulated in various cancers (Fig. 1A). We downloaded datasets for 12 colon tissues and 70 CRC tissues from the Oncomine database and analyzed NCAPG expression levels. NCAPG expression levels were over-expressed in CRC (Fig. 1B). In addition, we evaluated NCAPG expression levels in 60 pairs of CRC tissues and paracancerous normal tissues by qRT-PCR. NCAPG mRNA levels were substantially increased in CRC tissues compared to adjacent normal tissues (Fig. 1C). Furthermore, NCAPG mRNA and protein levels in CRC cell lines HCT116, SW480, and HT29 were assessed and found to be up-regulated compared to the colon epithelial cell line NCM460 (Fig. 1D–F).

### Overexpression of NCAPG may be correlated with poor prognosis in CRC patients

**Fig. 2:**
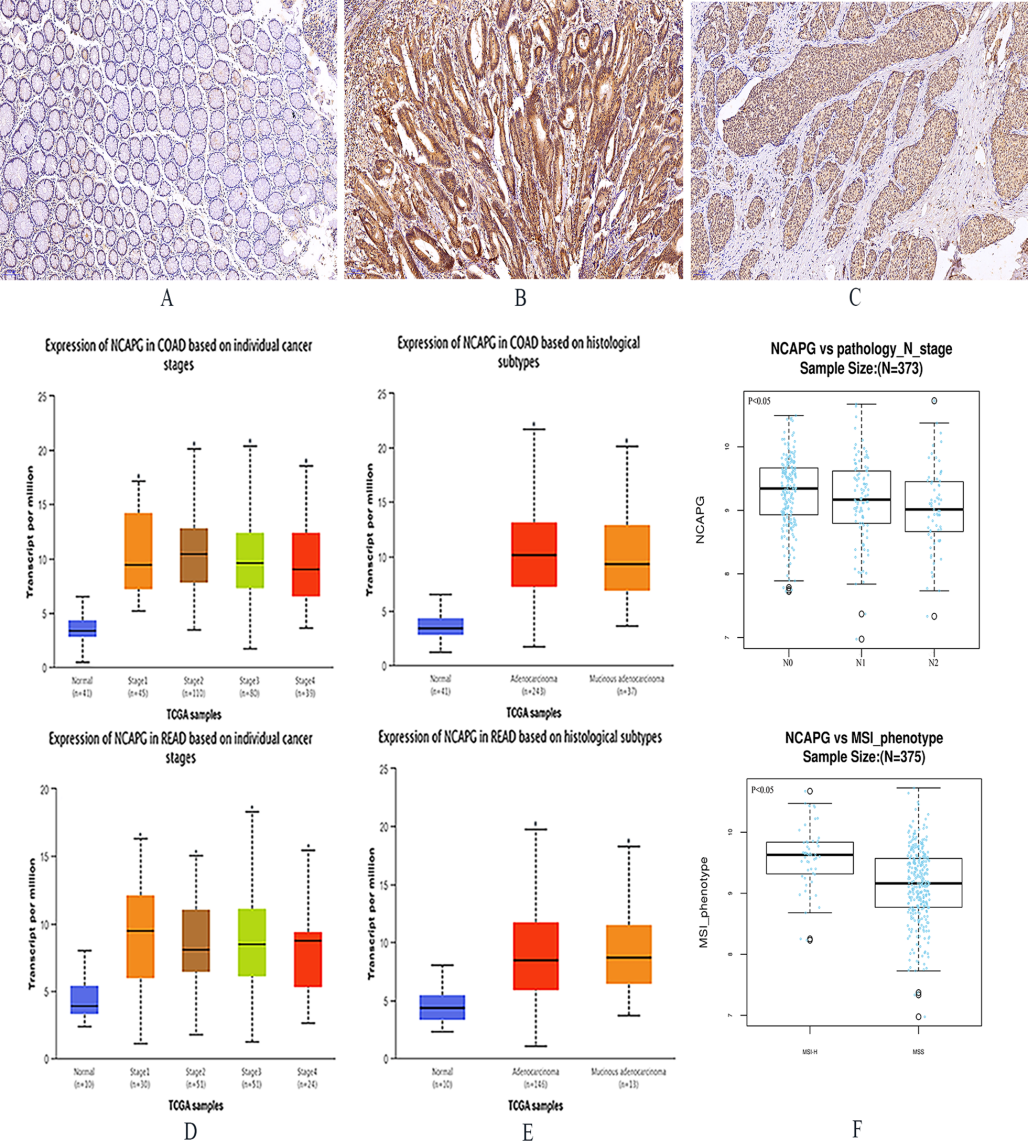
The expression of NCAPG protein is up-regulated in CRC, which is poor prognosis in CRC patients. **A** Adjacent tissues. **B** CRC tissues (endochylema). **C** CRC tissue (cell nucleus). **D**, **E** The UACLAN database identified and validated the relationship between NCAPG expression and clinicopathological characteristics. **F** Correlation of NCAPG expression with N stage and microsatellite instability phenotype. *P < 0.05

We measured the protein expression levels of NCAPG in 91 pairs of CRC tissue samples by immunohistochemistry, the results were as follows: the positive expression rate of NCAPG in CRC tissues and adjacent tissues were 73.56% (n = 68/91) and 28.42% (n = 27/91) respectively. The expression difference of NCAPG in tumor tissues and adjacent tissues were statistically significant (P < 0.0001). NCAPG was positively expressed in both the nucleus and nuclear plasma of CRC tissues (Fig. 2A–C). The positive expression levels of NCAPG were associated with differentiation levels, lymph metastasis, tumor, node (TNM), metastasis, and vascular invasion in patients (P < 0.05), but not with tumor site, tumor size, and preoperative CEA levels (Table 1). Moreover, bioinformatics analysis confirmed that the expression levels of NCAPG was correlated with TNM stage (P < 0.05), histological subtypes (P < 0.05), and N stage (P < 0.05) (Fig. 2D–E). Additionally, the expression levels of NCAPG were higher in CRC patients with microsatellite instability-high (MSI-H) compared to those with microsatellite stable (MSS) (Fig. 2F).

**Table 1:** Association between NCAPG expression and clinicopathological characteristics of CRC patients (n = 91)

Features	n	NCAPG expression		χ^2^	P value
		Low (n = 47)	High (n = 44)		
Gender					
Male	50	23	27	1.418	0.164
Female	41	24	17		
Age					
≤ 60	32	16	16	0.054	0.495
> 60	59	31	28		
Site					
Colon	54	29	25	0.225	0.397
Rectum	37	18	19		
Size (cm)					
≤ 5	63	32	31	0.060	0.493
> 5	28	15	13		
Differentiation					
High	3	3	0	16.608	< 0.001
Moderate	70	42	28		
Low	18	2	16		
T stage					
T1–T2	27	16	11	0.890	0.238
T3–T4	64	31	33		
Lymph metastasis					
No	49	32	17	7.930	< 0.05
Yes	42	15	27		
pTNM					
I + II	48	32	16	9.175	< 0.05
III + IV	43	15	28		
Vascular invasion					
No	52	34	18	9.167	< 0.05
Yes	39	13	26		
Perineural invasion					
No	60	34	26	1.776	0.133
Yes	31	13	18		
Preoperative CEA					
Negative	52	28	24	0.235	0.393
Positive	39	19	20		

P < 0.05 was statistically significant

### NCAPG overexpression and knockdown efficiency

**Fig. 3:**
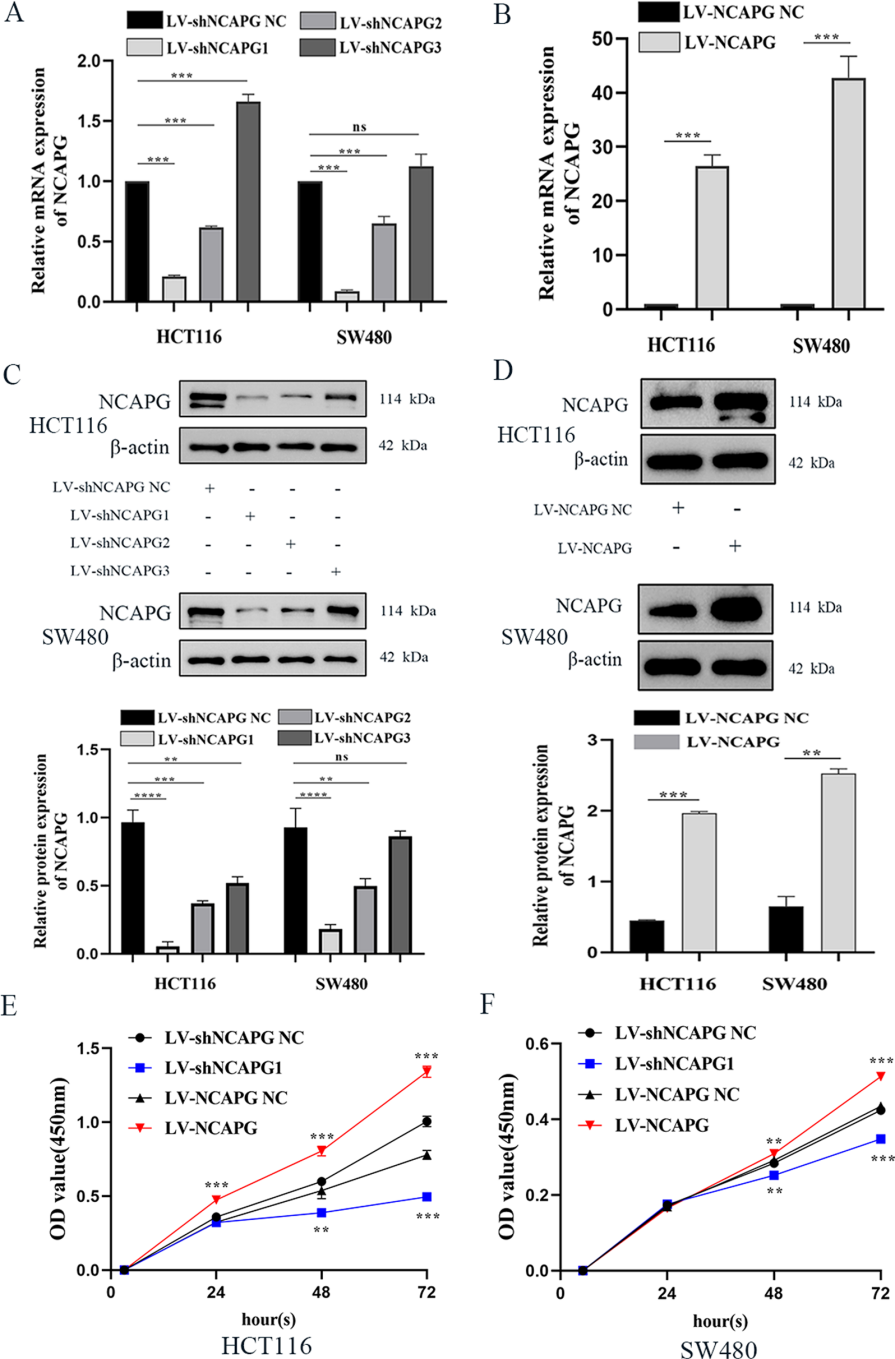
NCAPG affects CRC cell lines proliferation upon silencing or overexpression. **A**, **B** The mRNA of HCT116 and SW480 cell lines infected by lentivirus with knockdown or overexpression of NCAPG were detected by qRT-PCR. **C**, **D** NCAPG expression upon NCAPG knockdown or overexpression was measured by Western blotting in HCT116 and SW480 cell lines. **E**, **F** CCK-8 assay was performed to estimate HCT116 and SW480 cell lines proliferative ability upon NCAPG silencing or overexpression. *P < 0.05, **P < 0.01 and ***P < 0.001

We measured the expression levels of NCAPG knockdown or overexpression mRNA and protein in Lentivirus-constructed HCT116 and SW480 cells by by qRT-PCR and western blotting. The mRNA and protein levels of NCAPG in HCT116 and SW480 cells transducted with LV-shNCAPG1 were significantly lower compared to cells transfected with LV-shNCAPG NC (Fig. 3A, C). Furthermore, mRNA and protein levels of NCAPG in HCT116 and SW480 cells transduced with LV-NCAPG were higher compared to cells transduced with LV-NCAPG NC. This demonstrated that we successfully knocked down and overexpressed NCAPG in two CRC cell lines (Fig. 3B, D).

### NCAPG regulates the proliferation, migration and invasion of CRC Cells

**Fig. 4:**
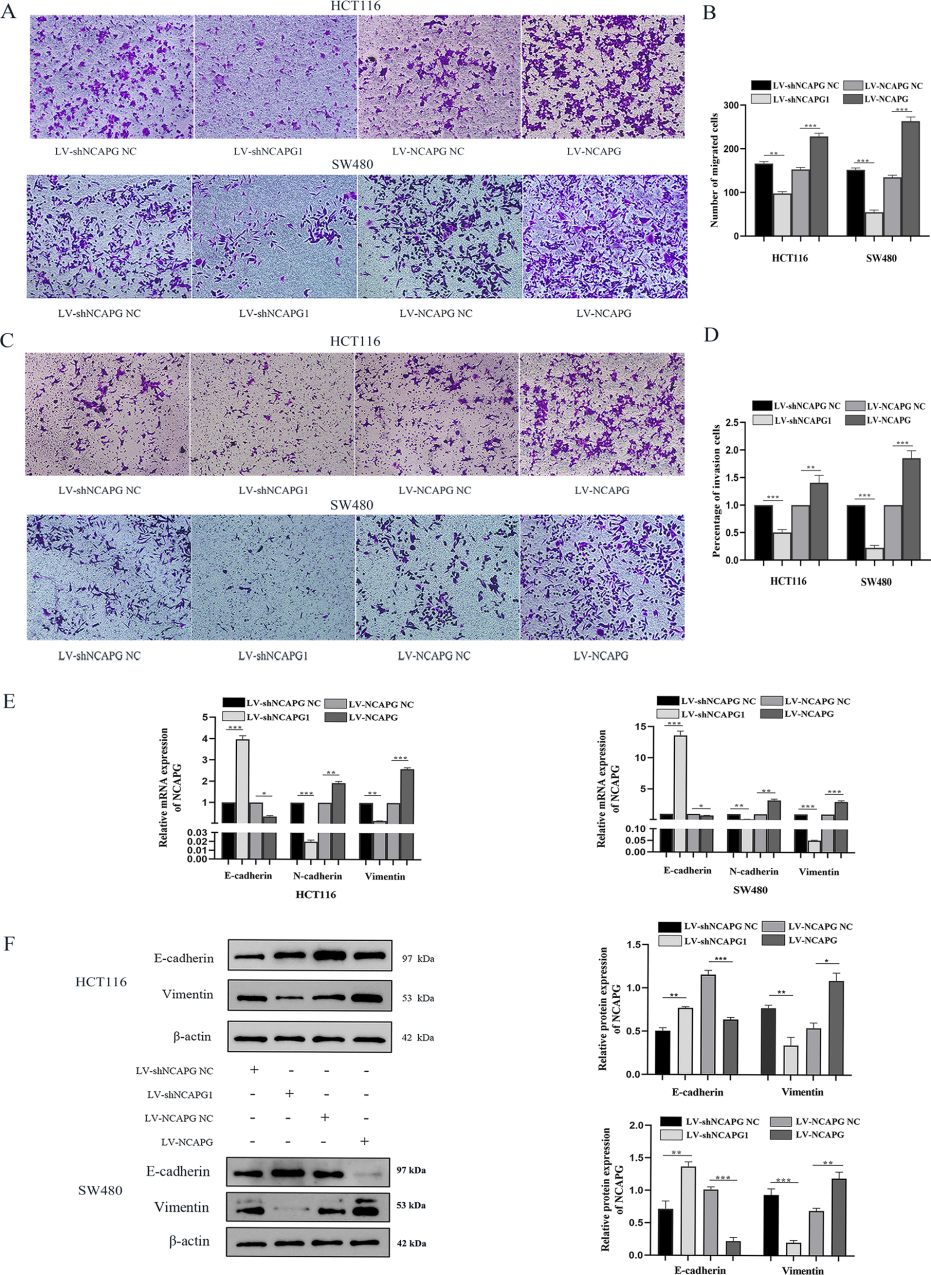
NCAPG modulates CRC cell lines aggressiveness and metastasis. **A** Transwell assay detected migration of HCT116 and SW480 cell lines upon NCAPG silencing or overexpression. **B** Histogram of the migrated cell numbers per microscopic field. **C** Transwell assay detected invasion of HCT116 and SW480 cell lines upon NCAPG silencing or overexpression. **D** Histogram of the invasive cell numbers per microscopic field. **E** Detect the effects of silencing or overexpression of NCAPG on EMT in HCT116 and SW480 cell lines by qRT-PCR. **F** Western blot detected the effect of NCAPG on EMT of HCT116 and SW480 cell lines. *P < 0.05, **P < 0.01 and ***P < 0.001

CCK-8 assays demonstrated that NCAPG silencing notably reduced the proliferation of HCT116 and SW480 cells at 24 h, 48 h, and 72 h compared to cells transduced with LV-shNCAPG NC. Conversely, NCAPG overexpression led to an increase in proliferation of HCT116 and SW480 cells at 24 h, 48 h, and 72 h compared to cells transduced with LV-NCAPG NC (Fig. 3E, F). To elucidate the role of NCAPG on migration and invasion ability, we performed transwell assays using HCT116 and SW480 cells that overexpressed or knocked down the expression levels of NCAPG. As shown in Fig. 4A, B, the number of migrated cells in HCT116 and SW480 transduced with LV-shNCAPG1 was lower compared to cells transduced with LV-shNCAPG NC. Conversely, cells transduced with LV-NCAPG had increased migration ability compared to control cells. In addition, the invasion ability of HCT116 and SW480 cells was dramatically increased after transduction with LV-NCAPG but markedly decreased in cells transduced with LV-shNCAPG1 (Fig. 4C, D). These results strongly indicated that NCAPG could increase the migration and proliferation of CRC cells.

### NCAPG modulates EMT in CRC cell lines

To determine whether silencing or overexpression of NCAPG in CRC cells modulates EMT, we measured mRNA and protein expression levels of EMT-related genes in lentivirus-transfected HCT116 and SW480 cells by RT-qPCR and western blotting. As shown in Fig. 4E, F, cells transfected with LV-shNCAPG1 had lower Vimentin mRNA and protein expression levels and higher mRNA and protein levels of E-cadherin compared to HCT116 and SW480 cells transduced with LV-shNCAPG NC. Conversely, cells transduced with LV-NCAPG had a twofold increase in Vimentin mRNA levels and lower than 0.5-fold of E-cadherin mRNA levels compared to control cells. The expression levels of these EMT-associated proteins were consistent with their corresponding mRNA levels. Taken together, NCAPG plays an important role in modulating EMT in CRC cell lines.

### Effects of NCAPG silencing or overexpression on Wnt/β-catenin signaling pathway in CRC cell lines

**Fig. 5:**
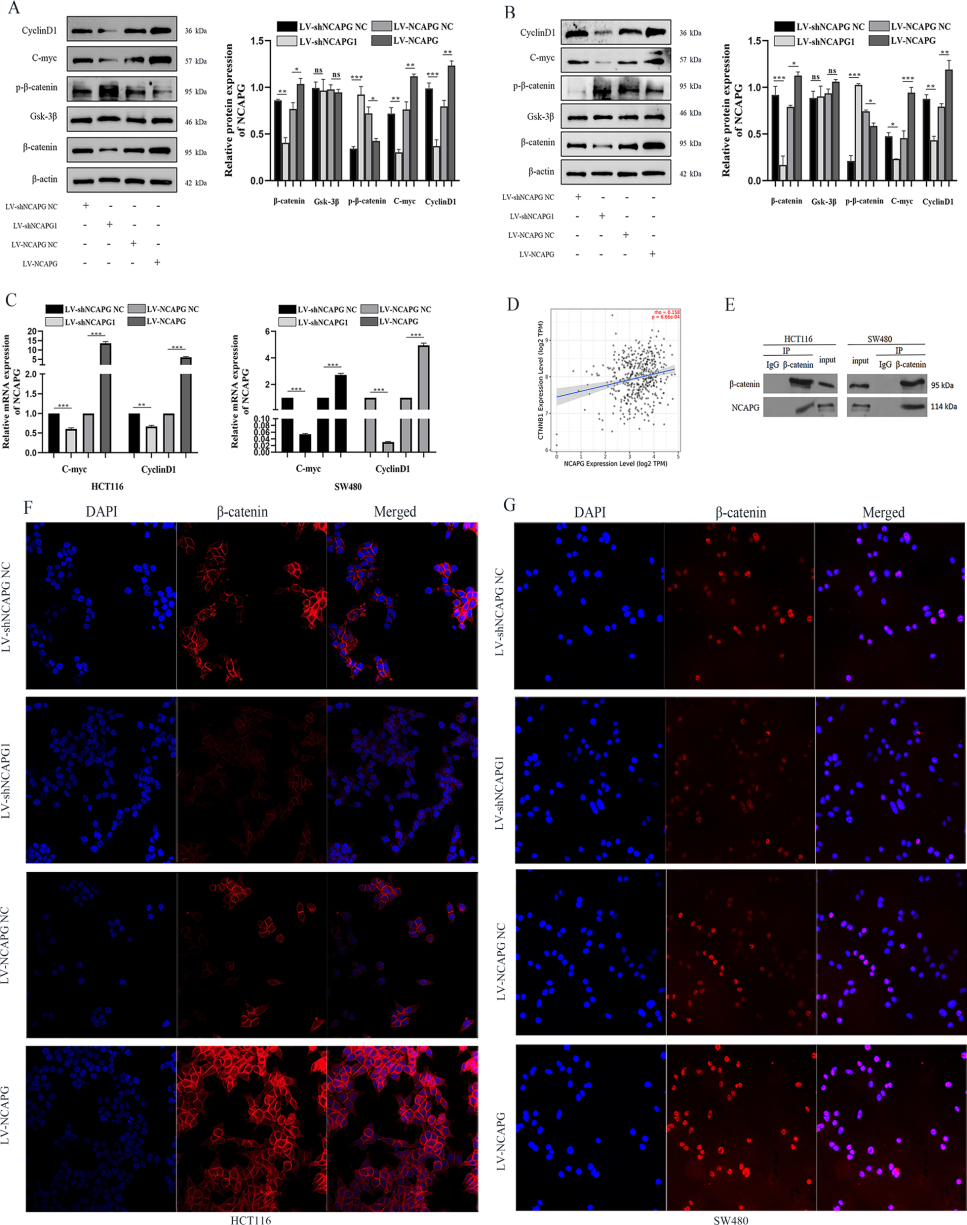
NCAPG may activate Wnt/β-catenin signaling pathway by binding to β-catenin in CRC cell lines. **A**, **B** Western blotting assessed the expression of Wnt/β-catenin signaling pathway related proteins upon NCAPG silencing or overexpression in HCT116 and SW480 cell lines. **C** The expression of Wnt/β-catenin signaling pathway target factors were examined in HCT116 and SW480 cell lines by qRT-PCR. **D** The expression of NCAPG is correlated with CTNNB1 in CRC. **E** The interaction between NCAPG and β-catenin was obtained by CO-IP. **F** The effects of silencing or overexpression of NCAPG on the localization of β-catenin in HCT116 and SW480 cell lines. *P < 0.05, **P < 0.01 and ***P < 0.001

To determine the mechanism of how NCAPG affects the Wnt/β-catenin signaling pathway in CRC cells, we measured protein expression levels of critical members of the Wnt/β-catenin signaling pathway, i.e. GSK3β, β-catenin, and p-β-catenin and their target genes, c-myc and cyclin D1. HCT116 and SW480 cells transduced with LV-NCAPG induced the activation of the Wnt/β-catenin pathway, as shown by the increased levels of β-catenin, c-myc, and cyclin D1 and reduced levels of p-β-catenin compared to cells in the control group (Fig. 5A, B). Conversely, NCAPG silencing showed the opposite trend. However, GSK-3β protein expression levels remained unchanged when NCAPG was silenced or overexpressed. We then determined whether NCAPG silencing could affect the targets of the Wnt/β-catenin signaling pathway, i.e., c-myc and cyclin D1. The mRNA levels of c-Myc and cyclin D1 in cells transduced with LV-shNCAPG1 were reduced compared to cells transduced with LV-shNCAPG NC (Fig. 5C). These findings demonstrate that NCAPG is involved in the activation of the Wnt/β-catenin signaling pathway, which subsequently affects proliferation and invasion of CRC.

### NCAPG activates Wnt/β-catenin signaling pathway by binding to β-catenin in CRC cell lines

A significant correlation in expression levels was observed for NCAPG and CTNNB1 in the TIME2.0 database (Fig. 5D). To identify the mechanism by which NCAPG regulates the Wnt/β-catenin signaling pathway, we performed CO-IP assays on endogenous NCAPG and β-catenin in CRC cells. Immunoprecipitation and western blot analysis demonstrated the presence of NCAPG protein in complexes pulled down using β-catenin antibodies in HCT116 and SW480 cells (Fig. 5E). This indicated that NCAPG may be bound to β-catenin either directly or indirectly. Additionally, immunofluorescence was used for sub-localization detection of β-catenin protein in lentivirus transfected cells. The β-catenin protein in the nucleus was more abundant in HCT116 and SW480 cells after NCAPG overexpression. Conversely, silencing NCAPG led to decreased β-catenin protein in the nucleus (Fig. 5F, G). These results further demonstrated that NCAPG facilitated the biological functions and EMT in CRC cells by activating the Wnt/β-catenin signaling pathway.

## Discussion

Colorectal cancer accounts for the second leading cause of mortality among cancers worldwide and causes a serious burden to the health care industry. Invasion and metastasis are the prime cause for poor prognosis in patients with CRC [27]. About 25% of patients with CRC are in the advanced stage when first diagnosed, and more than half succumb to the disease due to metastasis and recurrence [28, 29]. Although early diagnosis and treatment of CRC have improved, patient prognosis is still poor [30]. Hence advances must be made for the early diagnosis of patients. Understanding the signaling pathways, biological functions, and mechanisms leading to CRC is essential.

In this study, we demonstrated the biological function and underlying mechanism of NCAPG in CRC. Analysis of the Oncomine database showed that NCAPG expression was increased in CRC tissues compared to adjacent colon epithelial tissues. We then demonstrated that NCAPG expression was increased in CRC tissues and cell lines. Silencing and overexpression of NCAPG affected proliferation, migration and invasion in opposing manners. Furthermore, we demonstrated that NCAPG modulated EMT in CRC cell lines. NCAPG silencing or overexpression regulated the Wnt/β-catenin signaling pathway. Specifically, NCAPG activated the Wnt/β-catenin signaling pathway by binding to β-catenin in CRC cells.

DNA condensation is a crucial step during the initiation of mitosis, and lectin plays a vital role in this process. The association between NCAPG and tumors has been demonstrated extensively, however, its role in CRC has not been elucidated. By analyzing RNA-sequencing data based on TCGA, Xiao et al. [31] showed NCAPG to be over-expressed in 93% of tumor samples from lung squamous cell carcinoma, 74% for hepatocellular carcinoma, and 69% for breast cancer. High NCAPG expression levels are a prognostic marker for lung adenocarcinoma, and its high expression was negatively correlated with overall patient survival time [12]. It has been reported that NCAPG knockdown combined with temozolomide could inhibit the proliferation of pediatric high-grade gliomas [32]. In this study, we noticed that knockdown of NCAPG in CRC cells had lower proliferation rates compared to control CRC cells transfected with LV-shNCAPG NC. In addition, NCAPG silencing markedly decreased migration and invasion ability compared to control cells. This strongly indicated that NCAPG was important for invasion and metastasis of CRC. We additionally demonstrated that NCAPG increased the invasiveness and metastasis of CRC by overexpressing NCAPG in CRC cells. Wu et al. [33] NCAPG may promote EMT process by activating TGF-β signaling pathway, and then induce the occurrence of lung adenocarcinoma. To support this observation, we overexpressed or silenced NCAPG and measured EMT-related markers. After NCAPG overexpression in CRC cells, mRNA and protein expression levels of epithelial marker E-cadherin were reduced, while the mesenchymal marker Vimentin was increased. This suggested that NCAPG participated in modulating EMT in CRC cells.

Knockdown of NCAPG has been shown to regulate tumor proliferation, cell cycle and apoptosis through multiple signaling pathways [34–36]. Zhang et al. [11] demonstrated that NCAPG regulates cardia adenocarcinoma progression both in vivo and in vitro via the PI3K/AKT signaling pathway. Previous studies have also demonstrated that the Wnt/β-catenin signaling pathway was closely associated with poor prognosis, invasion, and metastasis of CRC. Our results demonstrated that NCAPG silencing reduced β-catenin as well as downstream targets of the Wnt/β-catenin signaling pathway, i.e., c-myc and cyclin D1 in CRC cells, while overexpression of NCAPG had the opposite effect. Additionally, we noticed that NCAPG silencing could promote the phosphorylation of β-catenin in CRC cells but this effect was reversed by NCAPG overexpression. Silencing of NCAPG was also shown to reduce endometrial cancer cell growth by inactivating the Wnt/β-catenin signaling pathway. Additionally, Jiang et al. showed that NCAPG induced trastuzumab resistance by regulating the SRC/STAT3 signaling pathway in HER2-positive breast cancer [10]. However, the specific mechanism by which NCAPG activates the Wnt/β-catenin pathway requires additional studies. We found a correlation of NCAPG expression with CTNNB1 in the TIMER2.0 database. Using CO-IP and IF assays, we found that NCAPG interacted with β-catenin physically and demonstrated its role in the activation of the Wnt/β-catenin signaling pathway in CRC. NCAPG silencing or overexpression resulted in reduced or increased levels of β-catenin in the nucleus.

## Conclusion

Our study identified that NCAPG was overexpressed in CRC tissues and cells and was associated with poor prognosis in CRC patients. NCAPG induced proliferation, migration, invasion, and EMT by activating the Wnt/β-catenin signaling pathway in CRC cells. Our results demonstrated the underlying mechanism by which NCAPG modulates CRC progress and metastasis. NCAPG could be a potential biomarker and/or therapeutic target for CRC.

## Data Availability

The data generated or analyzed in this study have all been published in this manuscript.

## Abbreviations

CRC: Colorectal cancer
NCAPG: The condensation complex gene non-SMC condensin I complex subunit G
EMT: Epithelial–mesenchymal transition
CO-IP: Co-immunoprecipitatio
IF: Immunofluorescence
TCGA: The Cancer Genome Atlas
